# Epidermal Inclusion Cysts of the Head and Neck: A One-Year Tertiary Care Experience With Emphasis on the Periauricular Region and Review of Literature

**DOI:** 10.7759/cureus.92250

**Published:** 2025-09-13

**Authors:** Tejaswi Mishra, Swaha Panda, Madhu Chaudhary, Gulistan Bano, Dheeraj Kumar, Suji PS, Bhartendu Bharti, KSBS Krishna Sasanka, Anila Sinha, Pradosh Kumar Sarangi

**Affiliations:** 1 Otolaryngology - Head and Neck Surgery, All India Institute of Medical Sciences, Deoghar, Deoghar, IND; 2 Pathology, All India Institute of Medical Sciences, Deoghar, Deoghar, IND; 3 Radiodiagnosis, All India Institute of Medical Sciences, Deoghar, Deoghar, IND

**Keywords:** benign tumor, epidermal inclusion cyst, epidermoid cyst, fnac, head and neck, otolaryngology, periauricular region, recurrence, subcutaneous nodule, surgical excision

## Abstract

Introduction: Epidermal inclusion cysts (EICs) are common benign lesions of epidermal origin, often presenting as soft, fluctuant subcutaneous swellings. The head and neck region is one of the commonest sites of its occurrence, with the periauricular area being a distinct subset because of its anatomical and cosmetic considerations. This study highlights the clinical profile, management, and outcomes of periauricular EICs at a tertiary care center.

Materials and methods: A retrospective observational study was conducted over one year, from February 2024 to February 2025, in the Department of Otorhinolaryngology at a tertiary care center in Eastern India. Twenty-two FNAC-confirmed EIC cases in the head and neck region were analyzed, and among them, six cases located in the periauricular area were discussed in detail.

Results: The patient cohort ranged from nine to 51 years (median 21.5 years), with equal gender distribution. Periauricular EIC (n = 6, 27.3%) was the most common. All patients underwent complete surgical excision with no recurrence observed during the follow-up period of three to six months. Cosmetic outcomes were generally satisfactory in all cases.

Conclusions: Periauricular EICs, while benign, require careful clinical evaluation due to their location and aesthetic implications. Complete surgical excision remains the treatment of choice, with low recurrence and excellent prognosis.

## Introduction

Epidermal inclusion cysts (EICs) are common, benign lesions that originate from epidermal tissue and are characterized by a keratin-filled sac lined with stratified squamous epithelium [1]. They are frequently encountered in clinical dermatology and may appear on various parts of the body. Typically, they present as palpable, subcutaneous nodules with a soft, fluctuant consistency. Histologically, they contain keratinous debris within a cavity lined by stratified squamous epithelium, including a granular layer [2]. Among the common anatomical sites, the head and neck region is one of the frequently involved sites, and of these occurrences, the periauricular area constitutes approximately one-tenth [3]. This location, adjacent to the external ear and its underlying cartilaginous structures, poses specific clinical and surgical considerations. Here, we present our institute's experience with six cases of periauricular EIC among 22 head and neck EIC patients, along with the management strategies employed.

## Materials and methods

This retrospective study was conducted in the department of Otorhinolaryngology and Head and Neck Surgery at a tertiary care center in Eastern India over a period of one year, from February 2024 to February 2025. The study included fine-needle aspiration cytology (FNAC)-confirmed cases of EICs presenting as head and neck masses in patients of all age groups. FNAC yielding other pathologies and recurrence cases were excluded.

Clinical data were collected and analyzed with respect to presenting symptoms, anatomical location, clinical characteristics, surgical management, and recurrence status. Follow-up duration ranged from a minimum of three months to a maximum of six months. A total of 22 cases were reviewed, of which six involved the periauricular region. These periauricular cases are detailed in the Results section. Informed consent was obtained from all patients before inclusion in the study.

Data was collected in a Microsoft Excel sheet (Microsoft Corp., Redmond, WA, USA), and descriptive analysis of data, including mean, median, and range, was done using SPSS Statistics (IBM Corp., IBM SPSS Statistics for Windows. Armonk, NY: IBM Corp.).

## Results

A total of 22 patients were recruited for this study. Their ages ranged from nine to 51 years, with a median age of 21.5 years and an interquartile range of 10. The cohort comprised an equal distribution of sexes, with 11 males (50%) and 11 females (50%) patients, resulting in a male-to-female ratio of 1:1. A majority of the patients (n = 15, 68.2%) resided in rural dwellings. In contrast, the remaining seven patients (31.8%) were from urban settings.

**Figure 1:**
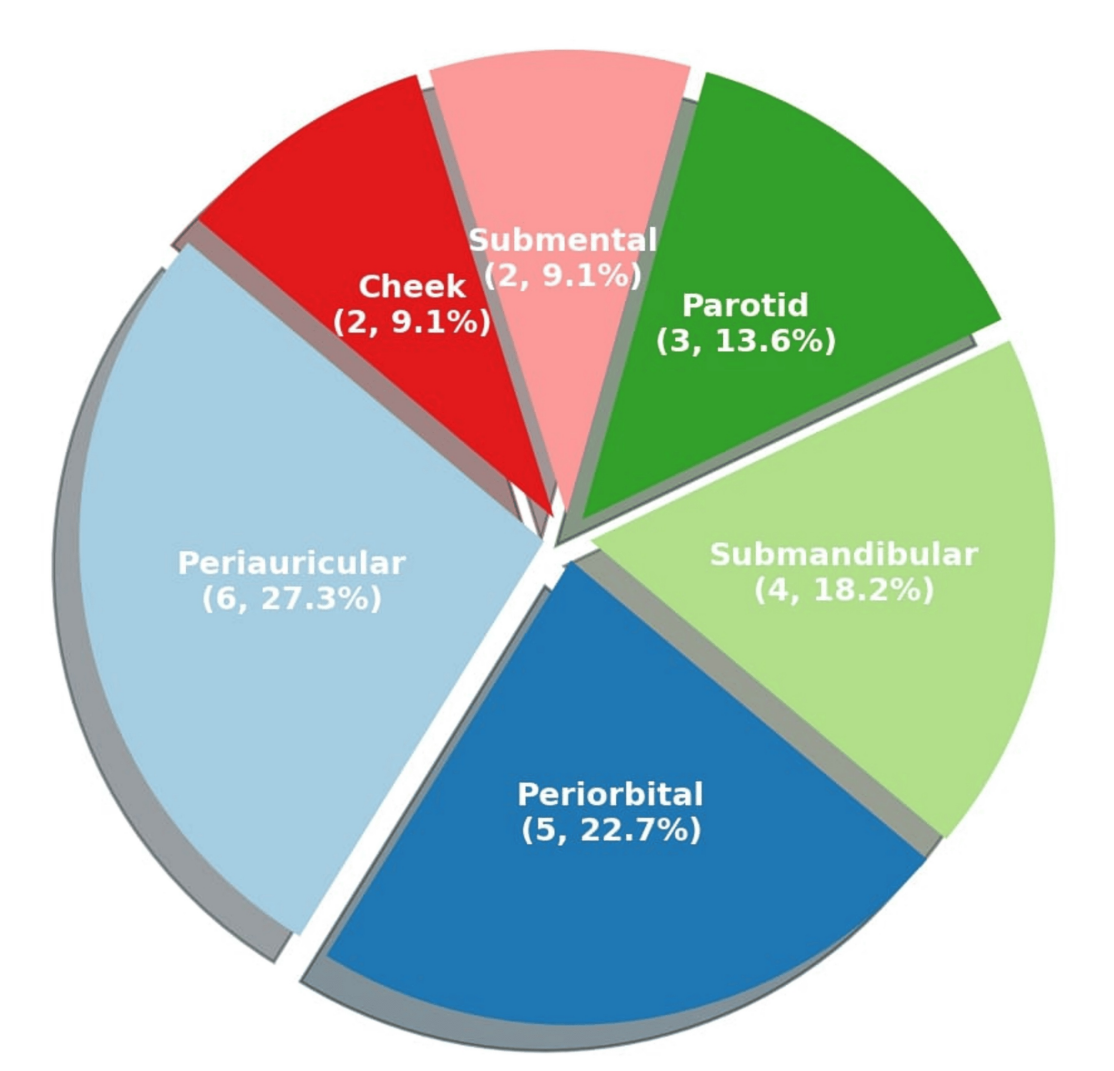
Pie chart showing the percentage occurrence of epidermal inclusion cysts at various sites in the head and neck region

Based on anatomical distribution, the cysts exhibited a right-sided predominance, with 13 patients (59.1%) presenting with lesions on the right side, while nine patients (40.9%) presented with lesions on the left. To determine the laterality of submental cysts, an imaginary line was drawn from the symphysis menti to the hyoid bone, dividing the submental triangle. Both of the cases in question had swellings located more towards the right side of this triangle and were therefore classified as right-sided lesions. Regarding the region-wise distribution of the cysts, the periauricular region was the most commonly affected, noted in 6 patients (27.3%). The periorbital region was affected in five patients (22.7%), the submandibular region in four patients (18.2%), and the parotid region in three patients (13.6%). Lesions were also observed in the submental and cheek regions, with two patients (9.1%) affected in each location (Figure 1). During the follow-up period, none of the patients demonstrated any evidence of lesion recurrence, suggesting a favorable clinical outcome post-surgical excision. Distribution of EICs across different head and neck sites in relation to patient sex, lesion laterality, place of residence, and recurrence status is shown in Table 1.

**Table 1:** Distribution of epidermal inclusion cysts by site, sex, laterality, residence, and recurrence n: number

Site	Sex		Side		Residence		Recurrence	
	Male (n (%))	Female (n (%))	Right (n (%))	Left (n (%))	Rural (n (%))	Urban (n (%))	Yes (n (%))	No (n (%))
Periorbital	2 (40.0)	3 (60.0)	3 (60.0)	2 (40.0)	4 (80.0)	1 (20.0)	0 (0.0)	5 (100.0)
Cheek	1 (50.0)	1 (50.0)	1 (50.0)	1 (50.0)	1 (50.0)	1 (50.0)	0 (0.0)	2 (100.0)
Submental	1 (50.0)	1 (50.0)	2 (100.0)	0 (0.0)	1 (50.0)	1 (50.0)	0 (0.0)	2 (100.0)
Parotid	2 (66.7)	1 (33.3)	0 (0.0)	3 (100.0)	2 (66.7)	1 (33.3)	0 (0.0)	3 (100.0)
Submandibular	1 (25.0)	3 (75.0)	2 (50.0)	2 (50.0)	3 (75.0)	1 (25.0)	0 (0.0)	4 (100.0)
Periauricular	4 (66.7)	2 (33.3)	5 (83.3)	1 (16.7)	4 (66.7)	2 (33.3)	0 (0.0)	6 (100.0)

**Figure 2:**
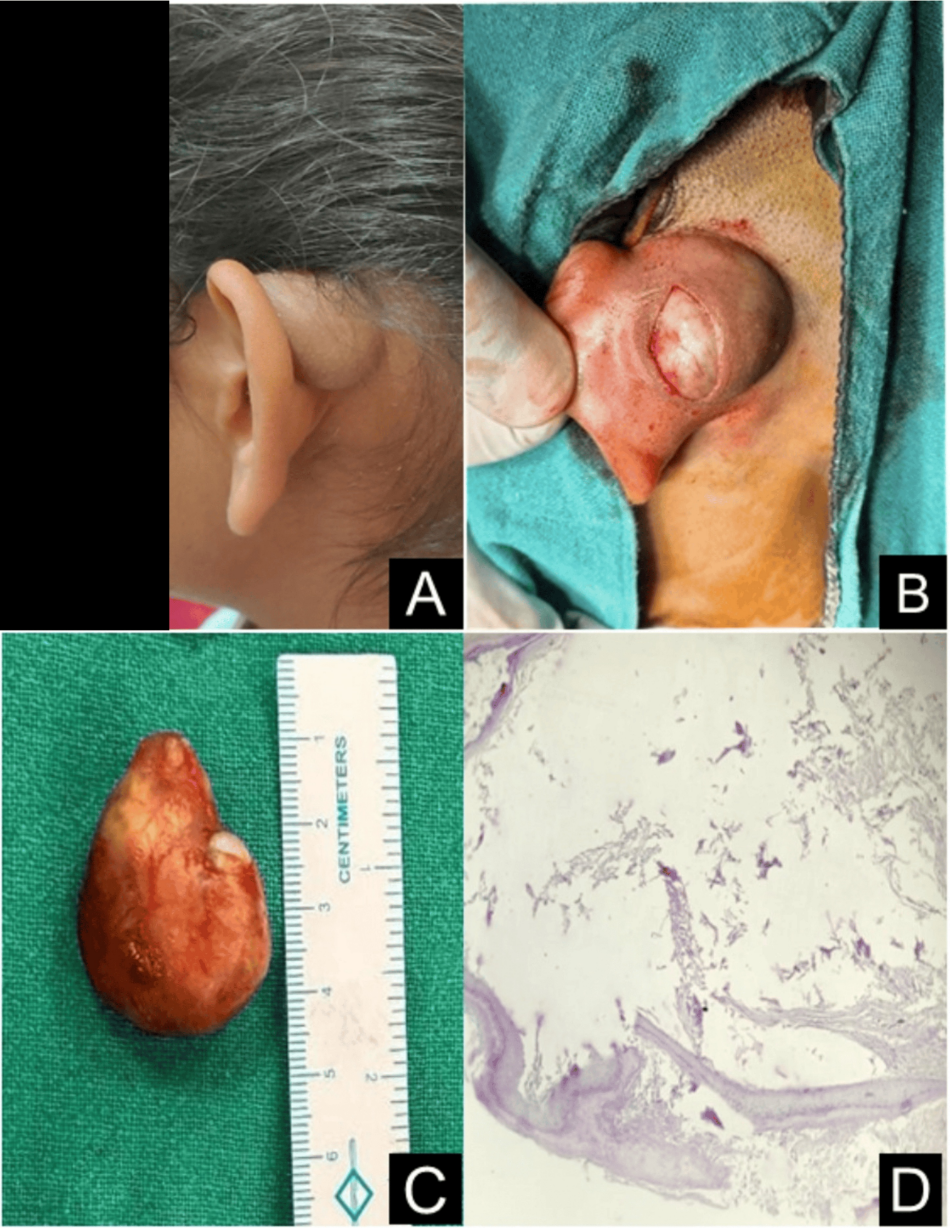
(A) Right ear epidermal inclusion cyst on the upper medial surface of the pinna extending to the postauricular region. (B) intraoperative view showing the curvilinear incision used for excision. (C) excised specimen in toto measuring approximately 3.5 × 2.5 cm. (D) histopathology revealing stratified squamous epithelium with a granular layer and keratin debris

Among the six cases of periauricular EICs, one case involved a 21-year-old female presenting with a cyst on the upper medial surface of the pinna, extending to the postauricular region. The patient reported a five-year history of insidious-onset, gradually progressive swelling. Clinical examination revealed a mobile, non-tender, fluctuant mass measuring approximately 3.5 × 3 cm. The cyst was excised entirely surgically, and the patient was doing well at six-month follow-ups, with no evidence of recurrence (Figure 2).

**Figure 3:**
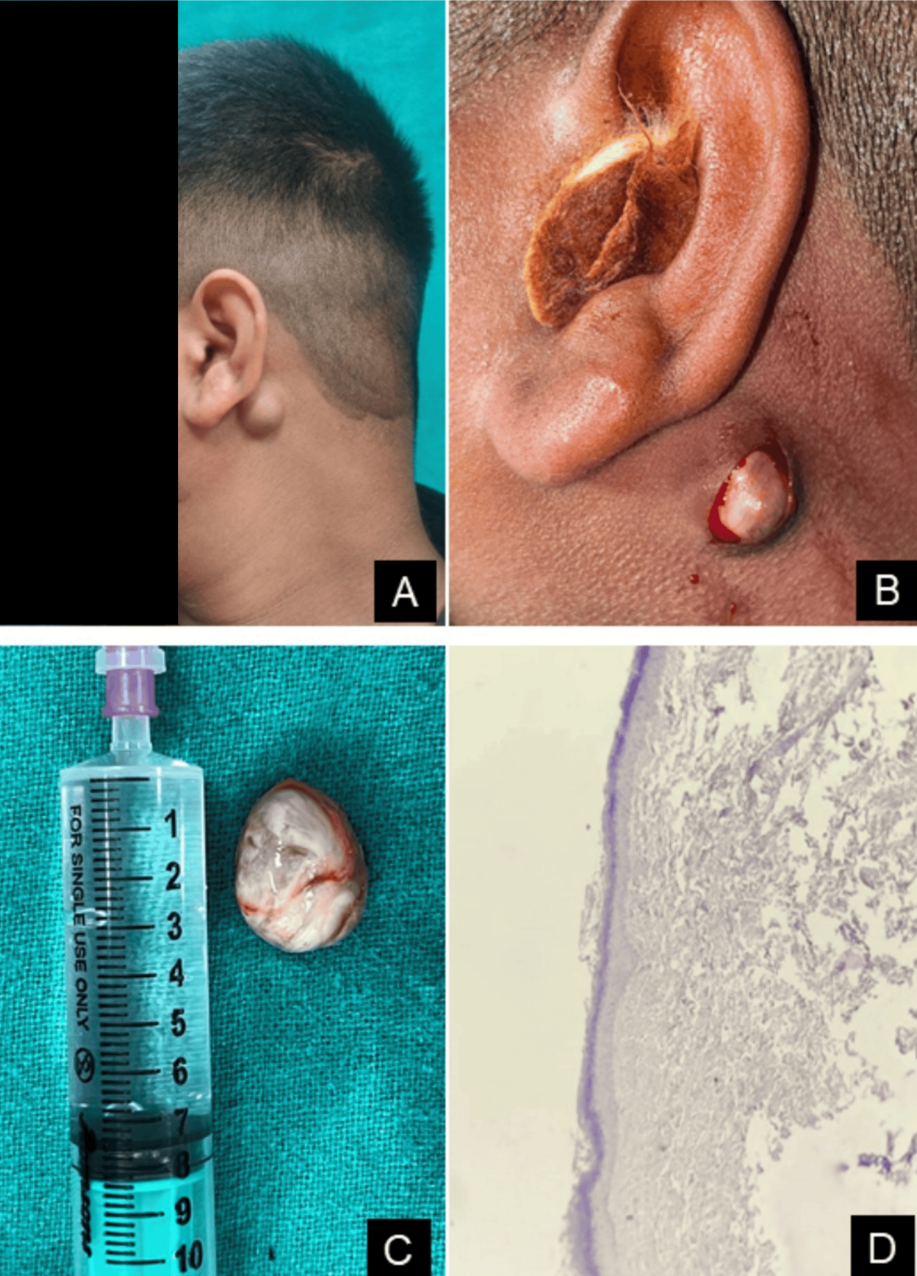
(A) Left post-auricular epidermal inclusion cyst. (B) Intraoperative view. (C) Excised specimen measuring approximately 2.5 × 2 cm. (D) Histopathology showing stratified squamous epithelium with abundant keratin flakes

**Figure 4:**
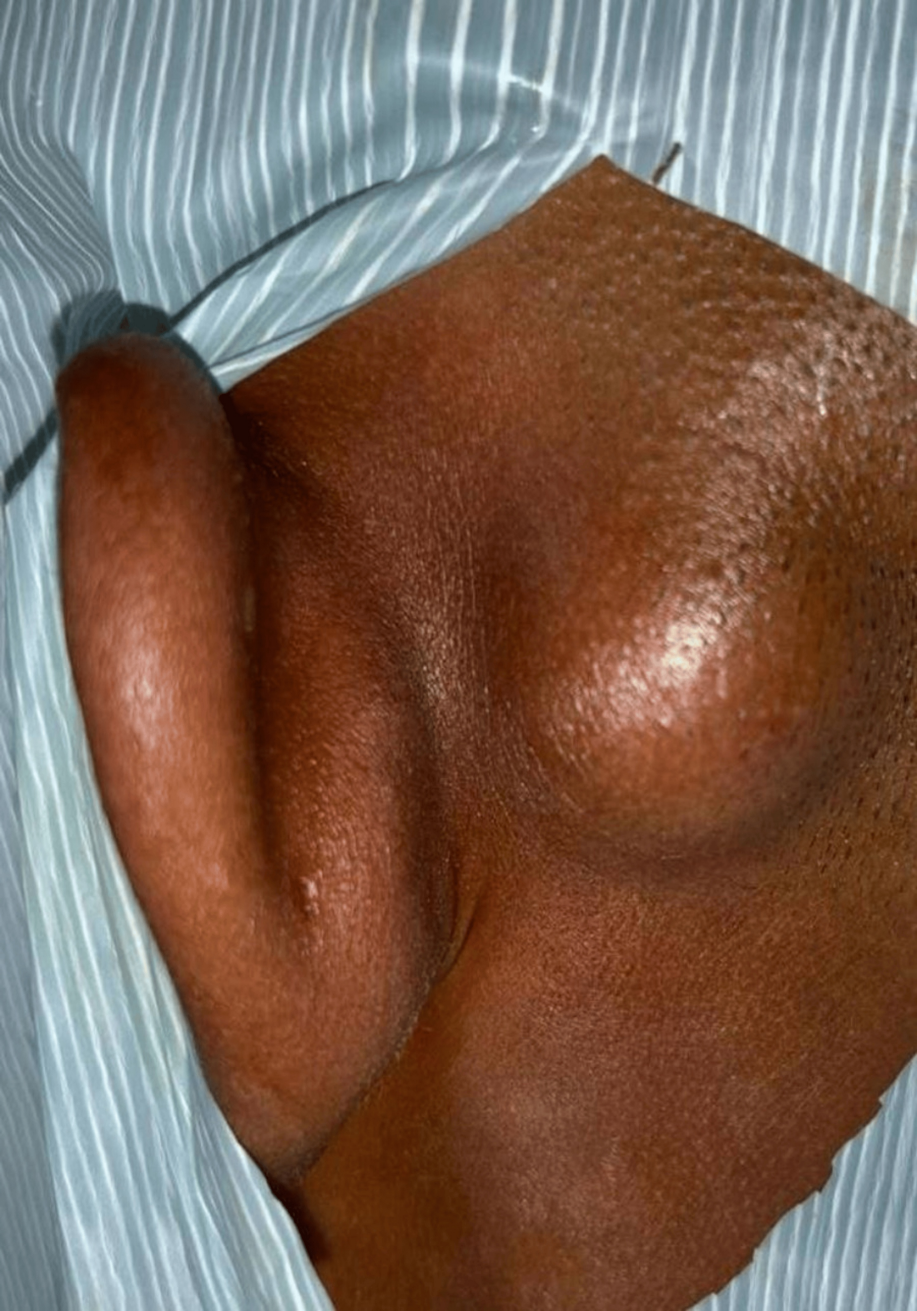
Epidermal inclusion cyst in the right postauricular region measuring approximately 3 × 3 cm

**Figure 5:**
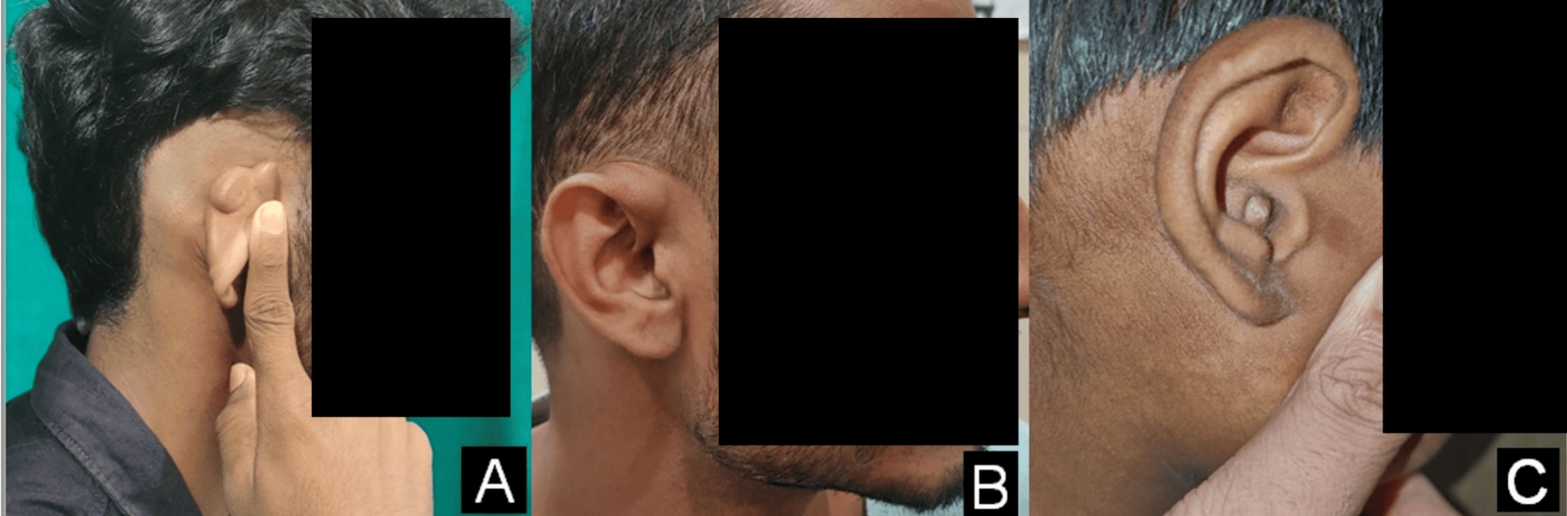
Epidermal inclusion cyst involving (A) the right medial surface of the pinna, (B) the superior aspect of the ascending helix, and (C) the outer external auditory canal

Two patients (33.3%) had postauricular EIC (Figures 3-4). One of them, a 23-year-old male, presented with a painless, slowly enlarging swelling behind the left ear over the past three years. On examination, the lesion was soft, freely mobile, and non-tender, measuring around 2.5 × 2 cm. The lesion was excised in toto following careful dissection along the cyst wall. The patient demonstrated no recurrence at a five-month follow-up (Figure 3). The other patient with postauricular swelling was a 51-year-old female with the onset of swelling for about one year. Clinical examination showed a 3 × 3 cm soft, fluctuant, and mobile swelling, which was surgically excised in toto with a healthy scar without any signs of recurrence till 6 months of follow-up (Figure 4). The remaining three patients included a 21-year-old male, a 25-year-old male, and an 11-year-old boy, presenting with EICs located on the medial surface of the pinna, the superior aspect of the ascending helix, and the outer external auditory canal, respectively (Figure 5). All three patients underwent complete surgical excision of the cysts. No recurrence was observed in any of these cases during a three-month follow-up period. All five (100%) periorbital EICs were localized to the medial canthus region.

## Discussion

Background

Among the various benign dermatological conditions, EICs are characterized by the formation of a subcutaneous sac lined by epidermal tissue that fills with keratinous material. They are also referred to in medical literature as epidermoid cysts, epidermal cysts, infundibular cysts, inclusion cysts, and keratin cysts, reflecting the shared characteristics of their origin and content [1]. Histologically, the defining feature of an EIC is a cystic cavity encased by stratified squamous epithelium, including a granular layer, and the accumulation of keratinous debris within the cyst due to normal turnover of the epidermal cells [2].

Among the frequent locations for EICs, the head and neck region is one of the most common sites for their development. Specifically, the periauricular area, which encompasses the skin and underlying tissues immediately surrounding the external ear, can also be affected by these benign lesions. The occurrence of EICs in this region introduces a unique set of clinical considerations. Due to their anatomical proximity to the external auditory canal and the cartilaginous structure of the ear, their presentation and surgical management approach can vary. Additionally, due to the visible nature of this location, the cosmetic outcome of any intervention is often a significant concern for patients.

Definition and terminology

EICs are cystic structures that are lined by stratified squamous epithelium, leading to the increased production and retention of keratin [4]. The absence of skin adnexal structures, such as hair follicles and sebaceous glands within the wall of the epidermal cyst, is the key histological feature that distinguishes EICs from dermoid cysts [5]. This precise histological definition, particularly the absence of these skin appendages, is crucial for differentiating periauricular EICs from other cystic lesions that may occur in the same region, such as branchial cleft cysts or dermoid cysts, having different embryological origins and potential to exhibit distinct clinical behaviors. Additionally, it is important to understand that the term "sebaceous cyst," which is commonly used interchangeably, is a misnomer in this context. True sebaceous cysts usually originate from the sebaceous glands. They are filled with sebum, being a relatively uncommon condition in comparison to EICs, which are filled with keratin and are a far more prevalent condition [1]. Thus, when a clinician encounters a cyst in the periauricular region, the differential diagnosis includes several possibilities, and only the histological examination of the excised cyst is the definitive method to rule them out and confirm the presence of an EIC.

Epidemiology and prevalence

EICs usually occur more commonly in adults in comparison with the extremes of age, with the highest incidence observed in individuals between the ages of 20 and 40 years. However, our study showed a broader age spectrum, which ranged from nine to 51 years. Across various available literature, a slight male predominance has often been reported irrespective of their location on the body [2]. However, our clinical observation was not suggestive of any sex-based predilection.

The head and neck region is a well-known region for EIC formation, accounting for approximately 60% of all EICs [6]. Within the broader category of head and neck EICs, the periauricular area is one of the common sites for their occurrence. Although epidemiological data focusing exclusively on periauricular EICs are limited, one study that examined facial epidermal cysts reported that about 10% are located around the ear region [3]. However, in our experience, periauricular EICs accounted for around 27% of head and neck EICs.

Pathogenesis

Due to the abnormal migration and subsequent proliferation of epidermal cells within the dermis, the epidermal cells, which generally constitute the outermost protective layer of the skin, begin to multiply and differentiate within a confined space in the dermis, leading to the formation of the wall of the cyst [6]. This cyst wall is characterized by a lining of stratified squamous epithelium, which typically includes a granular layer, a histologically significant feature [2]. The accumulation of keratinous material, which is often described as having a cheesy or paste-like consistency and sometimes emitting a foul odor due to the presence of breakdown products, is responsible for the formation of the palpable mass that is characteristic of these cysts [1]. Since there is an increased risk for traumatic implantation in the periauricular area, possibly resulting from common practices like ear piercing or minor local injuries, clinicians need to elicit such a history when evaluating patients presenting with these cysts in this particular location, as this information can provide valuable insight into the likely origin of the lesion.

Clinical presentation

Periauricular EICs present as a noticeable subcutaneous nodule or mass and are described as slow-growing and may remain asymptomatic for a considerable period. On physical examination, they are usually a dome-shaped lump that feels soft to the touch and is freely mobile beneath the skin when palpated. The size of these cysts can vary, ranging from just a few millimeters in diameter to several centimeters in larger cases [1]. The skin over the cyst is usually intact and normal in color; however, in some cases, there can be a subtle bluish tint or a slight discoloration of the skin [4].

In most of the patients, these lesions do not cause any pain or significant symptoms, but their infection and inflammation can lead them to become tender to the touch, painful, red, and swollen. Rupture of the cyst can rarely occur, leading to the release of its contents, which are described as a whitish-yellow, thick, and often foul-smelling material, and can cause local irritation and inflammation of the surrounding tissues. In the specific context of the periauricular region, larger cysts, even if they are not inflamed or infected, can cause a sensation of pressure or general discomfort simply due to their presence in a relatively confined anatomical location.

Since the periauricular region is a prominent area on the face, even relatively smaller EICs in this region can be a cosmetic concern for the patients, making them seek medical advice and treatment. An interesting case report described an unusual presentation of an infected preauricular cyst that was not associated with a preauricular sinus, serving as a reminder for clinicians to be aware of potential variations from the more routine clinical presentations of these lesions [7]. Due to the aesthetic implications of a visible lump, this may influence a patient's decision to seek treatment, even for an asymptomatic periauricular EIC, thereby highlighting the cosmetic outcome of any intervention as a crucial factor in the overall management plan.

Diagnostic methods

The initial diagnosis of a periauricular EIC is typically made through a thorough and careful clinical examination. The characteristic appearance of a subcutaneous nodule, which is mobile on palpation, is a key clinical indicator.

Many imaging techniques can be employed in diagnosing EIC, as these can provide valuable supplementary information in certain situations apart from diagnosing routine cases. Among these, ultrasound is a non-invasive imaging modality that can be used to confirm the cystic nature of the periauricular lesion, helping to evaluate its size and the regularity of its borders, as well as to look for any associated signs of inflammation or potential complications. On ultrasound, an intact EIC typically appears as a well-circumscribed, round, or oval structure that is either anechoic (appearing black, indicating fluid content) or hypoechoic (appearing darker than surrounding tissues) [8]. In certain situations, such as an unusually large cyst, an atypical or deep position of the cyst, or if there is clinical suspicion of involvement of deeper anatomical structures or the presence of other underlying pathology, a computed tomography (CT) scan may be indicated. A CT scan can provide detailed cross-sectional anatomical information, helping to differentiate the cyst from other types of lesions that may occur in the periauricular region, as well as assess for involvement of the underlying bony structures of the skull, particularly if the cyst is situated very close to the bone [9]. On the other hand, magnetic resonance imaging (MRI) is another advanced imaging technique that can be used when detailed information about the contents of the cyst and its precise relationship to the surrounding soft tissues in the periauricular area is needed. On MRI, EICs exhibit low to intermediate signal intensity on T1-weighted images and high signal intensity on T2-weighted images and may also demonstrate restricted diffusion on diffusion-weighted imaging sequences [10].

The definitive diagnosis of a periauricular EIC is ultimately confirmed through histopathological examination of the tissue following the surgical excision. In certain circumstances, when there is a need for a rapid preliminary assessment or to help differentiate a periauricular cystic lesion from other possibilities, FNAC can be performed. Cytological examination of the material aspirated from the cyst typically reveals a clear background containing numerous squamous cells, both with and without nuclei, along with fragments of keratinous debris [6]. While FNAC can provide suggestive evidence of an EIC, the definitive diagnosis usually requires the histopathological examination of the entire excised cyst.

Treatment options

When the periauricular EICs are small, asymptomatic, and not causing any functional impairment or cosmetic concerns, these can be observed without any immediate active intervention. However, if they become inflamed or infected, incision and drainage have to be done as an initial step in management to relieve the pressure and evacuate any purulent material that has accumulated within the cyst. However, incision and drainage alone do not address the underlying cyst wall; thus, the rate of recurrence following this approach is typically high [2]. Additionally, intralesional injection of corticosteroids directly into the cyst may be considered a treatment option for cysts that are symptomatic due to inflammation but do not show signs of active infection [1]. Although aspiration of the cyst contents using a needle aspiration can temporarily reduce the size of the cyst, the lesion recurs in due time. Minimally invasive techniques, such as the use of carbon dioxide laser or radiofrequency ablation, have been explored as potential treatment modalities for small epidermal cysts. However, their application in the periauricular area has not been documented in the literature. In specific situations, such as managing multiple small epidermal cysts known as milia, topical retinoid medications may be used [2]. Additionally, intralesional administration of hydrolytic enzymes has been reported as a successful minimally invasive treatment in a single case report of cheek EIC. Still, further, more comprehensive studies are needed to fully evaluate the efficacy and safety of this approach [11].

Complete surgical excision of the cyst is the most definitive and widely recommended treatment for periauricular EICs. The primary objective of surgical intervention is the complete removal of the entire cyst, including the cyst wall. This significantly reduces the chances of cyst recurrence in the future. Surgical excision is generally best performed when the cyst is not acutely inflamed or infected, as the presence of inflammation can make the surgical dissection more challenging and increase the likelihood of the cyst rupturing during the procedure.

When performing surgical excision of cysts in the periauricular region, the meticulous surgical technique is of paramount importance, not only to achieve successful removal of the cyst and prevent its recurrence but also to ensure a good cosmetic outcome and to avoid any inadvertent damage to nearby anatomical structures, such as the cartilaginous framework that gives the ear its shape and support. Given the cosmetic sensitivity of the periauricular region, when surgical excision is planned, the surgeon should prioritize surgical techniques that are known to minimize scarring, such as making incisions within natural skin creases and employing meticulous techniques for wound closure.

Recurrence and prognosis

The likelihood of a periauricular EIC recurring after complete surgical excision, where the entire cyst wall has been successfully removed, is generally quite low. In rare instances, a periauricular cyst may recur even after what appeared to be a complete excision, as highlighted in at least one reported case [9].

The overall prognosis for patients who develop periauricular EICs is excellent, as these lesions are benign in nature. Complete surgical removal of the cyst typically results in a permanent resolution of the condition. The occurrence of long-term complications is relatively uncommon if the cyst is managed appropriately and completely excised.

Limitations

Our study is an institutional perspective on periauricular EICs and is subject to several key limitations that constrain the generalizability of its findings. Firstly, it is a retrospective study in which patients were recruited based on our institutional medical records, which may be subject to information and selection bias. Our patient cohort of 22 cases and a detailed sub-cohort of six periauricular EICs represents a relatively small sample size, which limits the statistical power of our observations and makes our findings on patient demographics and anatomical distribution unique to our institution rather than broadly representative. Additionally, the follow-up period of three to six months is insufficient to definitively assess long-term recurrence rates for these typically slow-growing lesions. While no recurrences were observed in our cohort, the true incidence of recurrence may only become apparent with a more extended follow-up. Furthermore, our analysis was restricted to patients who underwent complete surgical excision, precluding a comparative evaluation of alternative management strategies such as minimally invasive techniques or nonsurgical approaches.

## Conclusions

EICs are commonly encountered benign lesions in the head and neck region, with the periauricular area emerging as a particularly frequent site in our institutional experience. The anatomical complexity and aesthetic importance of this region necessitate careful clinical evaluation and precise surgical planning. Our findings underscore the importance of considering EICs in the differential diagnosis of periauricular swellings and highlight the efficacy of complete surgical excision as a definitive treatment modality. Further multicentric studies with larger sample sizes and longer follow-ups are warranted to validate these observations and to better understand the etiopathogenesis, recurrence patterns, and optimal management strategies for periauricular EICs.
